# Safety and Pharmacokinetics of LHF-535, a Potential Treatment for Lassa Fever, in Healthy Adults

**DOI:** 10.1128/aac.00951-22

**Published:** 2022-10-31

**Authors:** Sean M. Amberg, Benjamin Snyder, Portia A. Vliet-Gregg, Eric J. Tarcha, Jeff Posakony, Kristin M. Bedard, Alison E. Heald

**Affiliations:** a Kineta, Inc., Seattle, Washington, USA; b Nucleus Network Pty Ltd., Melbourne, Victoria, Australia; c Alfred Health, Melbourne, Victoria, Australia; d University of Washington, Seattle, Washington, USA

**Keywords:** antiviral agents, antiviral pharmacology, Lassa fever

## Abstract

LHF-535 is a small-molecule antiviral currently under development as a therapeutic option to treat Lassa fever and other viral hemorrhagic fevers of arenavirus origin. The human safety and pharmacokinetics of LHF-535 were evaluated in two phase 1 trials in healthy volunteers. The first study was a double-blind, single ascending dose trial that evaluated weight-based oral doses ranging from 0.3 mg/kg in the first cohort to 40 mg/kg in the last cohort. The second study was a double-blind, multiple ascending dose trial that evaluated a 14-day oral dosing regimen, with three sequential cohorts receiving fixed doses of 450, 900, or 1,125 mg per day; the third cohort (1,125 mg/day) received a higher (loading) dose of 2,250 mg for the first dose. Each cohort in both studies consisted of eight participants randomized to either placebo (*n* = 2) or LHF-535 (*n* = 6). LHF-535 was well tolerated in both studies. Treatment-emergent adverse events were more frequent in placebo recipients than in LHF-535 recipients in both studies. LHF-535 exhibited rapid absorption, a long half-life, and exposures predicted to suppress viral replication.

## INTRODUCTION

Lassa fever is a viral hemorrhagic disease endemic to West Africa. While the true burden of the disease remains unclear, the most commonly cited numbers indicate several hundred thousand cases per year, with an estimated 5,000 deaths annually (1, 2). Lassa fever generates high mortality and is one of the highest known zoonotic spillover threats (3); a range of case fatality rates among hospitalized patients has been reported, with rates of 21 to 43% in Nigeria over a nearly 2-decade span (4) and a more recent report of 12% (5). Treatment for Lassa fever includes supportive care and intravenous ribavirin, a broad-spectrum antiviral. While there is historical evidence that supports the use of ribavirin to treat Lassa fever (6), recent analyses have challenged these assumptions and suggested that ribavirin may actually result in negative outcomes, particularly in milder cases (7, 8). Effective therapeutics for Lassa fever thus remain to be identified. LHF-535 is a small-molecule entry inhibitor that targets the viral envelope glycoprotein (9) and has exhibited a promising preclinical profile. Here, we evaluate the pharmacokinetics (PK) and safety of LHF-535 in a population of healthy adults.

## RESULTS

### Demographics.

A total of 72 participants were enrolled, 48 for the phase 1a single ascending dose (SAD) study (Table 1) and 24 for the phase 1b multiple ascending dose (MAD) study (Table 2), randomized to active LHF-535 or placebo at a ratio of 3:1. Each cohort included 8 participants. The demographic characteristics are presented in Table 1 (phase 1a SAD) and Table 2 (phase 1b MAD). The average age of participants was 27.4 years in the phase 1a SAD study and 30.5 years in the phase 1b MAD study. There were more male participants (64.6% in phase 1a and 58.3% in phase 1b) than female participants (35.4% in phase 1a and 41.7% in phase 1b). Most participants were white (75.0% in phase 1a and 87.5% in phase 1b); 22.9% of phase 1a and 8.3% of phase 1b participants were Asian, 2.1% of phase 1a participants were Black or African American, and 4.2% of phase 1b participants were American Indian or Alaska Native. The mean body mass index (BMI) was about 25 kg/m^2^ but ranged between a low of 22.8 kg/m^2^ (900 mg/day in the phase 1b MAD study) to a high of 28.3 kg/m^2^ (1,125 mg/day in the phase 1b MAD study) for group means.

**TABLE 1:** Demographic characteristics of the phase 1a SAD study population

Characteristic	Data by treatment group							
	LHF-535 (mg/kg)						All LHF-535	Placebo
	0.3	1.0	3.0	6.0	20	40		
No. of subjects	6	6	6	6	6	6	36	12
Age (yrs)								
Mean	29.0	25.8	28.2	30.3	21.3	27.8	27.1	28.4
SD	7.56	5.46	6.88	3.98	2.34	7.44	6.23	7.96
Median	28.0	26.0	26.0	30.5	20.5	30.0	27.5	26.0
Range	18–38	18–32	22–40	25–37	20–26	18–37	18–40	19–46
Gender (*n* [%])								
Male	5 (83.3)	5 (83.3)	3 (50.0)	3 (50.0)	5 (83.3)	4 (66.7)	25 (69.4)	6 (50.0)
Female	1 (16.7)	1 (16.7)	3 (50.0)	3 (50.0)	1 (16.7)	2 (33.3)	11 (30.6)	6 (50.0)
Race (*n* [%])								
Asian	2 (33.3)	0 (0.0)	1 (16.7)	2 (33.3)	3 (50.0)	2 (33.3)	10 (27.8)	1 (8.3)
Black or African American	0 (0.0)	0 (0.0)	1 (16.7)	0 (0.0)	0 (0.0)	0 (0.0)	1 (2.8)	0 (0.0)
White	4 (66.7)	6 (100)	4 (66.7)	4 (66.7)	3 (50.0)	4 (66.7)	25 (69.4)	11 (91.7)
Body wt (kg) at screening								
Mean	72.5	77.2	73.7	68.0	74.1	77.5	73.8	74.5
SD	18.43	14.94	14.86	9.39	17.47	16.60	14.76	13.25
Median	66.8	76.7	70.1	66.2	69.7	77.9	70.9	74.7
Range	55.3–100.3	54.3–100.5	57.0–91.9	56.7–80.9	55.6–105.4	56.7–94.1	54.3–105.4	54.2–105.3
BMI (kg/m^2^) at screening								
Mean	23.6	25.1	24.2	23.6	23.8	26.5	24.5	25.5
SD	3.05	4.65	4.44	1.71	5.51	4.71	4.04	3.40
Median	23.6	24.6	22.4	24.2	22.4	25.5	23.8	24.5
Range	19.8–27.3	19.0–32.8	20.3–30.2	20.3–25.2	19.0–33.6	21.6–32.8	19.0–33.6	21.2–32.5

**TABLE 2:** Demographic characteristics of the phase 1b MAD study population

Characteristic	Data by treatment group				
	LHF-535 (mg/kg)			All LHF-535	Placebo
	450	900	1,125^*a*^		
No. of subjects	6	6	6	18	6
Age (yrs)					
Mean	28.0	32.0	30.3	30.1	31.5
SD	3.69	3.69	7.45	5.21	12.39
Median	28.0	33.5	30.0	30.5	30.0
Range	22–33	25–35	23–39	22–39	19–50
Gender (*n* [%])					
Male	2 (33.3)	4 (66.7)	3 (50.0)	9 (50.0)	5 (83.3)
Female	4 (66.7)	2 (33.3)	3 (50.0)	9 (50.0)	1 (16.7)
Race (*n* [%])					
American Indian or Alaska Native	0 (0.0)	0 (0.0)	1 (16.7)	1 (5.6)	0 (0.0)
Asian	0 (0.0)	1 (16.7)	1 (16.7)	2 (11.1)	0 (0.0)
White	6 (100)	5 (83.3)	4 (66.7)	15 (83.3)	6 (100)
Body wt (kg) at screening					
Mean	69.0	70.6	86.0	75.2	77.7
SD	11.38	9.50	10.00	12.49	17.54
Median	68.3	69.7	86.1	73.8	74.1
Range	56.4–88.3	57.3–85.5	74.1–99.6	56.4–99.6	53.3–105.2
BMI (kg/m^2^) at screening					
Mean	23.5	22.8	28.3	24.9	25.0
SD	2.78	1.99	3.60	3.69	3.81
Median	23.6	22.4	27.9	24.5	23.3
Range	20.2–27.3	20.3–25.7	24.5–34.2	20.2–34.2	22.0–29.8

a Participants in the 1,125-mg group received a loading dose of 2,250 mg once on day 1, followed by a maintenance dose of 1,125 mg/day on days 2 through 14.

### Safety.

There were three serious adverse events (SAEs) reported in one participant in the phase 1a SAD study; there were no SAEs reported for the phase 1b MAD study. The three SAEs were mild, asymptomatic events reported as telemetry findings of nonconducted atrial beats, sinus bradycardia, and a long sinus pause of about 8 to 9 s. The participant was a 24-year-old male with no past medical history and no concomitant medications or known allergies who received a single dose of 276 mg LHF-535 (3.0 mg/kg); the SAEs were observed 17 to 18 h after dosing, while the participant was asleep. The participant was asymptomatic with a normal physical examination, stable vital signs, and a normal electrocardiogram (ECG). Prior to dosing, the participant had telemetry findings of sinus bradycardia, one 2.8-s sinus pause, and one nonconducted atrial contraction that occurred around 4 h prior to dosing. Urgent safety laboratory results were unremarkable (troponin I, comprehensive metabolic panel [including blood urea nitrogen, electrolytes, and creatinine], and thyroid function test). The echocardiogram showed no structural abnormalities. The participant was hospitalized for 2 days for observation, during which time there were no noteworthy telemetry findings and the participant remained asymptomatic. All subsequent protocol-specified assessments through day 29 were normal. On day 12, the participant began 24-h Holter monitoring, during which there were no clinically significant findings. Diagnostic findings from the Holter monitoring were no atrial or ventricular arrhythmias, 3 nocturnal pauses of up to 2.1 s, and no symptoms reported.

The most frequent treatment-emergent adverse events (TEAEs) reported for the phase 1a SAD study by system organ class (Table 3) were gastrointestinal disorders, which were reported for 8 of 36 LHF-535 participants (22.2%) and 1 of 12 placebo participants (8.3%). The most common gastrointestinal disorder was diarrhea, occurring in 5 of 36 LHF-535 recipients (13.9%) and none of the 12 placebo participants. Overall, the frequency of TEAEs in the LHF-535 participants (41.7%) was similar to that for placebo (50.0%). There was no dose-related increase in TEAEs by system organ class or preferred term. Treatment-emergent diarrhea was only reported at higher doses (20 and 40 mg/kg) but was less frequent (16.7%) at the highest dose (40 mg/kg) than at 20 mg/kg (66.7%). A similar frequency was observed for TEAEs considered to be treatment related (36.1% for LHF-535 and 50.0% for placebo). The most frequent treatment-related TEAE was diarrhea (13.9% for LHF-535 and none for placebo), and the second most frequent treatment-related TEAE was headache (8.3% for LHF-535 and 25.0% for placebo).

**TABLE 3:** Treatment-emergent adverse events occurring in two or more subjects per system organ class in the phase 1a SAD study^*a*^

TEAE	No. (%) of participants with AE by treatment^*b*^							
	LHF-535 (mg/kg)						All LHF-535	Placebo
	0.3	1.0	3.0	6.0	20	40		
Any TEAE	2 (33.3)	1 (16.7)	3 (50.0)	1 (16.7)	6 (100)	2 (33.3)	15 (41.7)	6 (50.0)
Gastrointestinal disorders	0 (0.0)	0 (0.0)	1 (16.7)	0 (0.0)	5 (83.3)	2 (33.3)	8 (22.2)	1 (8.3)
Diarrhea	0 (0.0)	0 (0.0)	0 (0.0)	0 (0.0)	4 (66.7)	1 (16.7)	5 (13.9)	0 (0.0)
Nausea	0 (0.0)	0 (0.0)	1 (16.7)	0 (0.0)	0 (0.0)	1 (16.7)	2 (5.6)	0 (0.0)
Abdominal pain	0 (0.0)	0 (0.0)	0 (0.0)	0 (0.0)	1 (16.7)	0 (0.0)	1 (2.8)	0 (0.0)
Upper abdominal pain	0 (0.0)	0 (0.0)	1 (16.7)	0 (0.0)	0 (0.0)	0 (0.0)	1 (2.8)	0 (0.0)
Gastroesophageal reflux disease	0 (0.0)	0 (0.0)	0 (0.0)	0 (0.0)	1 (16.7)	0 (0.0)	1 (2.8)	0 (0.0)
Tongue ulceration	0 (0.0)	0 (0.0)	0 (0.0)	0 (0.0)	1 (16.7)	0 (0.0)	1 (2.8)	0 (0.0)
Mouth hemorrhage	0 (0.0)	0 (0.0)	0 (0.0)	0 (0.0)	0 (0.0)	0 (0.0)	0 (0.0)	1 (8.3)
Infections and infestations	0 (0.0)	1 (16.7)	1 (16.7)	1 (16.7)	1 (16.7)	1 (16.7)	5 (13.9)	2 (16.7)
Upper respiratory tract infection	0 (0.0)	0 (0.0)	1 (16.7)	1 (16.7)	1 (16.7)	1 (16.7)	4 (11.1)	2 (16.7)
Oral herpes	0 (0.0)	1 (16.7)	0 (0.0)	0 (0.0)	0 (0.0)	0 (0.0)	1 (2.8)	0 (0.0)
Nervous system disorders	1 (16.7)	0 (0.0)	0 (0.0)	0 (0.0)	2 (33.3)	1 (16.7)	4 (11.1)	3 (25.0)
Headache	1 (16.7)	0 (0.0)	0 (0.0)	0 (0.0)	2 (33.3)	1 (16.7)	4 (11.1)	3 (25.0)
Dizziness	0 (0.0)	0 (0.0)	0 (0.0)	0 (0.0)	1 (16.7)	0 (0.0)	1 (2.8)	0 (0.0)
Dysgeusia	0 (0.0)	0 (0.0)	0 (0.0)	0 (0.0)	0 (0.0)	1 (16.7)	1 (2.8)	0 (0.0)
Dizziness postural	0 (0.0)	0 (0.0)	0 (0.0)	0 (0.0)	0 (0.0)	0 (0.0)	0 (0.0)	1 (8.3)
Musculoskeletal and connective tissue disorders	1 (16.7)	0 (0.0)	1 (16.7)	0 (0.0)	0 (0.0)	1 (16.7)	3 (8.3)	1 (8.3)
Back pain	0 (0.0)	0 (0.0)	1 (16.7)	0 (0.0)	0 (0.0)	0 (0.0)	1 (2.8)	1 (8.3)
Musculoskeletal pain	1 (16.7)	0 (0.0)	0 (0.0)	0 (0.0)	0 (0.0)	0 (0.0)	1 (2.8)	0 (0.0)
Musculoskeletal stiffness	0 (0.0)	0 (0.0)	0 (0.0)	0 (0.0)	0 (0.0)	1 (16.7)	1 (2.8)	0 (0.0)
Cardiac disorders	1 (16.7)	0 (0.0)	1 (16.7)	0 (0.0)	0 (0.0)	0 (0.0)	2 (5.6)	0 (0.0)
Atrioventricular block	0 (0.0)	0 (0.0)	1 (16.7)	0 (0.0)	0 (0.0)	0 (0.0)	1 (2.8)	0 (0.0)
Sinus arrest	0 (0.0)	0 (0.0)	1 (16.7)	0 (0.0)	0 (0.0)	0 (0.0)	1 (2.8)	0 (0.0)
Sinus bradycardia	0 (0.0)	0 (0.0)	1 (16.7)	0 (0.0)	0 (0.0)	0 (0.0)	1 (2.8)	0 (0.0)
Ventricular tachycardia	1 (16.7)	0 (0.0)	0 (0.0)	0 (0.0)	0 (0.0)	0 (0.0)	1 (2.8)	0 (0.0)
General disorders and administration site conditions	0 (0.0)	0 (0.0)	1 (16.7)	0 (0.0)	0 (0.0)	0 (0.0)	1 (2.8)	1 (8.3)
Infusion site pain	0 (0.0)	0 (0.0)	1 (16.7)	0 (0.0)	0 (0.0)	0 (0.0)	1 (2.8)	0 (0.0)
Fatigue	0 (0.0)	0 (0.0)	0 (0.0)	0 (0.0)	0 (0.0)	0 (0.0)	0 (0.0)	1 (8.3)

a A treatment-emergent adverse event (TEAE) is defined as an AE that commenced on or after the time of study drug administration through study completion 28 days after dosing; subjects with multiple occurrences of a TEAE are only counted once per given system organ class and preferred term. AEs were coded to system organ class and preferred term using MedDRA version 21.0.

b Each LHF-535 treatment group comprised 6 participants (*N* = 36). The placebo group comprised 12 participants.

The most frequent TEAEs reported for the phase 1b MAD study by system organ class (Table 4) were general disorders and administration site conditions, occurring in 5 of 18 LHF-535 participants (27.8%) and 1 of 6 placebo participants (16.7%); these included a vessel puncture site bruise (3 LHF-535 participants and 1 placebo), an infusion site erythema (1 LHF-535 participant), and a medical device site reaction (1 LHF-535 participant), consisting of skin irritation at an ECG lead sticker. Overall, the frequency of TEAEs in LHF-535 participants (72.2%) was similar to that in the placebo group (83.3%). Treatment-related TEAEs were more frequent in placebo participants (50.0%) than in LHF-535 participants (16.7%). The most common treatment-related TEAE was headache, occurring in one LHF-535 participant (5.6%) and in one placebo participant (16.7%); the headache in the LHF-535 participant was at the lowest dose (450 mg/day).

**TABLE 4:** Treatment-emergent adverse events occurring in two or more subjects per system organ class in the phase 1b MAD study^*a*^

TEAE	No. (%) of participants with AE by treatment^*b*^				
	LHF-535 (mg/day)			All LHF-535	Placebo
	450	900	1,125^*c*^		
Any TEAE	4 (66.7)	4 (66.7)	5 (83.3)	13 (72.2)	5 (83.3)
General disorders and administration site conditions	1 (16.7)	1 (16.7)	3 (50.0)	5 (27.8)	1 (16.7)
Vessel puncture site bruise	0 (0.0)	1 (16.7)	2 (33.3)	3 (16.7)	1 (16.7)
Infusion site erythema	1 (16.7)	0 (0.0)	0 (0.0)	1 (5.6)	0 (0.0)
Medical device site reaction	0 (0.0)	0 (0.0)	1 (16.7)	1 (5.6)	0 (0.0)
Infections and infestations	2 (33.3)	1 (16.7)	1 (16.7)	4 (22.2)	1 (16.7)
Upper respiratory tract infection	2 (33.3)	1 (16.7)	1 (16.7)	4 (22.2)	1 (16.7)
Nervous system disorders	2 (33.3)	0 (0.0)	0 (0.0)	2 (11.1)	2 (33.3)
Headache	2 (33.3)	0 (0.0)	0 (0.0)	2 (11.1)	2 (33.3)
Dizziness postural	0 (0.0)	0 (0.0)	0 (0.0)	0 (0.0)	1 (16.7)
Gastrointestinal disorders	1 (16.7)	1 (16.7)	0 (0.0)	2 (11.1)	1 (16.7)
Toothache	0 (0.0)	1 (16.7)	0 (0.0)	1 (5.6)	0 (0.0)
Diarrhea	1 (16.7)	0 (0.0)	0 (0.0)	1 (5.6)	1 (16.7)
Nausea	0 (0.0)	1 (16.7)	0 (0.0)	1 (5.6)	0 (0.0)
Abdominal discomfort	0 (0.0)	0 (0.0)	0 (0.0)	0 (0.0)	1 (16.7)
Investigations	0 (0.0)	1 (16.7)	1 (16.7)	2 (11.1)	0 (0.0)
Electrocardiogram T-wave inversion	0 (0.0)	0 (0.0)	1 (16.7)	1 (5.6)	0 (0.0)
Blood creatine phosphokinase increased	0 (0.0)	1 (16.7)	0 (0.0)	1 (5.6)	0 (0.0)
Injury, poisoning and procedural complications	1 (16.7)	1 (16.7)	0 (0.0)	2 (11.1)	0 (0.0)
Contusion	1 (16.7)	0 (0.0)	0 (0.0)	1 (5.6)	0 (0.0)
Vascular access site pain	0 (0.0)	1 (16.7)	0 (0.0)	1 (5.6)	0 (0.0)
Skin and subcutaneous tissue disorders	0 (0.0)	2 (33.3)	0 (0.0)	2 (11.1)	0 (0.0)
Dermatitis contact	0 (0.0)	2 (33.3)	0 (0.0)	2 (11.1)	0 (0.0)
Respiratory, thoracic and mediastinal disorders	1 (16.7)	0 (0.0)	0 (0.0)	1 (5.6)	3 (50.0)
Dry throat	1 (16.7)	0 (0.0)	0 (0.0)	1 (5.6)	0 (0.0)
Nasal congestion	0 (0.0)	0 (0.0)	0 (0.0)	0 (0.0)	1 (16.7)
Oropharyngeal pain	0 (0.0)	0 (0.0)	0 (0.0)	0 (0.0)	2 (33.3)

a A treatment-emergent adverse event (TEAE) is defined as an AE that commenced on or after the first time of study drug administration through study completion 28 days following the final dose; subjects with multiple occurrences of a TEAE are only counted once per given system organ class and preferred term. AEs were coded to system organ class and preferred term using MedDRA version 22.0.

b Each LHF-535 treatment group comprised 6 participants (*N* = 18). The placebo group comprised 6 participants.

c For the 1,125-mg group, participants received a loading dose of 2,250 mg once on day 1, followed by a maintenance dose of 1,125 mg/day on days 2 through 14.

With one exception, all treatment-related TEAEs in both clinical trials were classified as mild (grade 1). The exception was observed in the phase 1a SAD, in which one participant reported moderate (grade 2) abdominal pain 5 days following a dose of 3.0 mg/kg. There were two TEAEs in the phase 1a SAD study that were classified as moderate (grade 2) but considered to be unrelated to treatment. One was a headache of moderate severity, beginning 11 days after a single dose of 20 mg/kg and resolving within 2 days, and the other was an upper respiratory tract infection of moderate severity, beginning 24 days after a single dose of 40 mg/kg and resolving within 7 days. There were also two moderate (grade 2) TEAEs in the phase 1b MAD study (one myalgia and one upper respiratory tract infection) that were considered to be unrelated to treatment; these were both in the placebo group. Across both studies, all TEAEs were transient and considered resolved by the final study visit. There was no clinically significant pattern of changes in hematology, coagulation, biochemistry, or urinalysis, there was no consistent change or trend of concern identified with vital signs, and there were no clinically significant ECG or physical examination findings observed.

### Pharmacokinetics.

**FIG 1:**
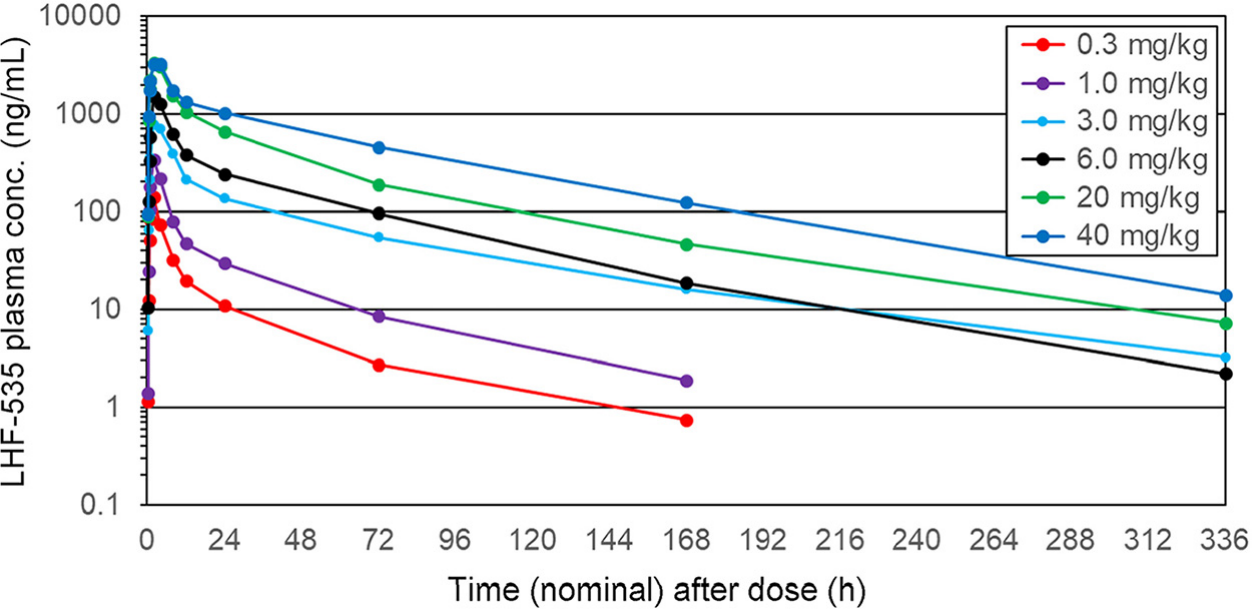
Mean plasma LHF-535 concentration in the phase 1a SAD study.

**FIG 2:**
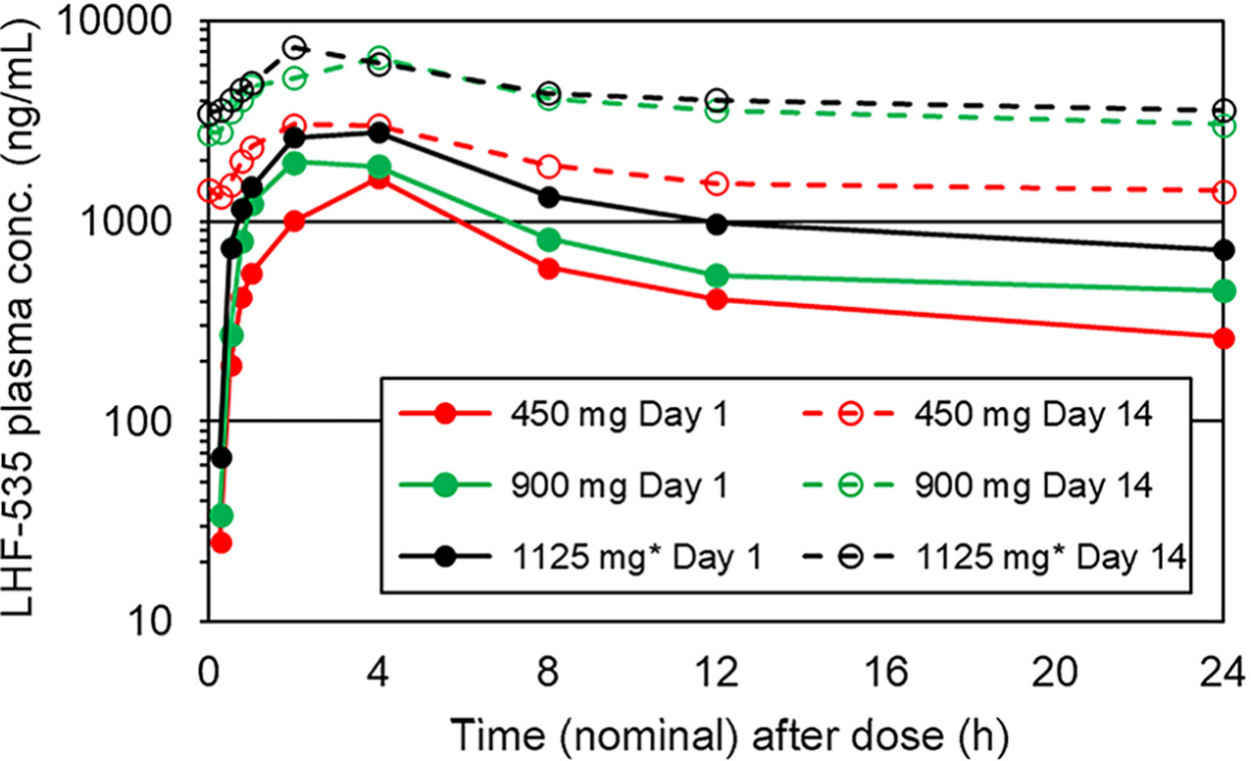
Mean plasma LHF-535 concentration in the phase 1b MAD study; overlay of days 1 and 14. * for 1125 mg group, participants received a loading dose of 2250 mg once on day 1, followed by a maintenance dose of 1125 mg/day on days 2 through 14.

**FIG 3:**
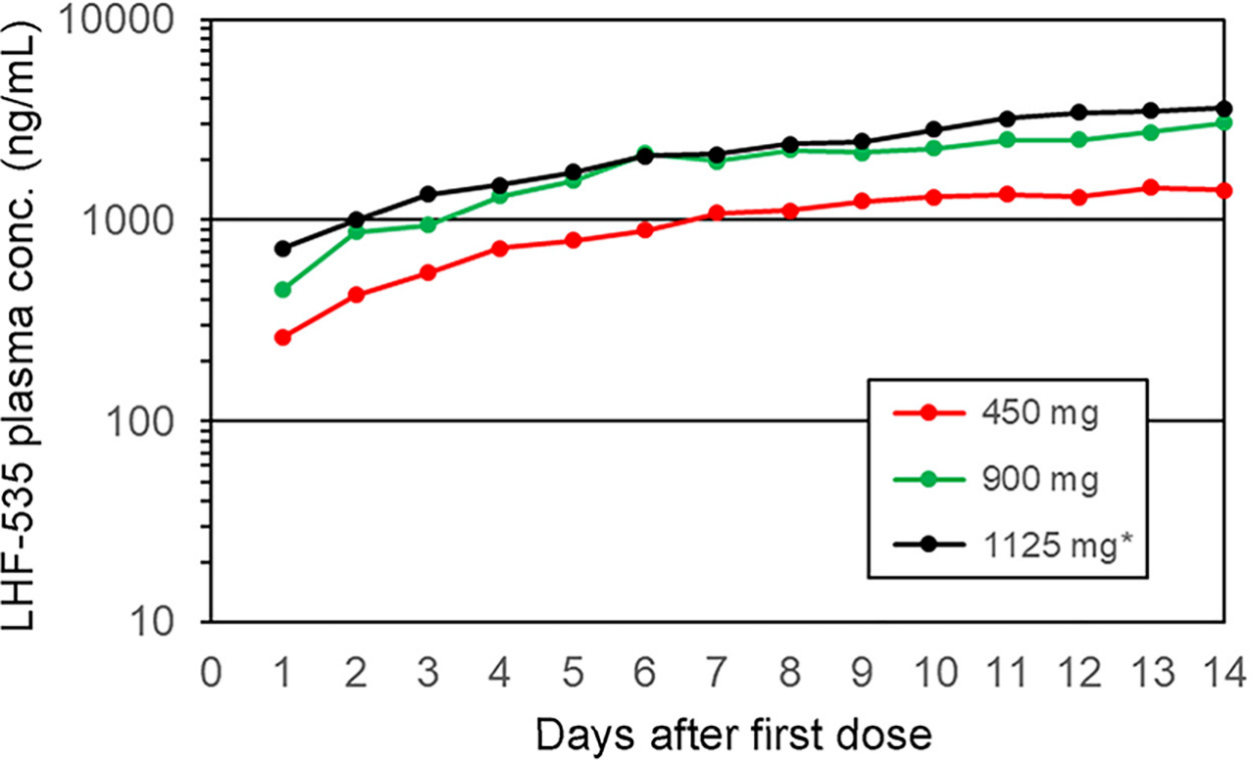
Mean trough plasma LHF-535 concentration in the phase 1b MAD study. Each point represents the mean concentration 24 h after the last dose and immediately prior to the next dose. * for 1125 mg group, participants received a loading dose of 2250 mg once on day 1, followed by a maintenance dose of 1125 mg/day on days 2 through 14.

LHF-535 is rapidly absorbed following oral administration, with a time to maximum concentration of drug in plasma (*T*_max_) of 2 to 4 h postdose (Fig. 1 and 2). Overall, the mean plasma levels increased with increasing doses. In the single-dose study (Table 5), the total exposure (area under the concentration-time curve [AUC]) increased with the dose in a nearly dose-proportional fashion, with slopes of 0.92 for the AUC extrapolated to the last dose (AUC_0–last_) and 0.91 for AUC_0–∞_. Increases in the maximum concentration of drug in serum (*C*_max_) were less than dose proportional across all doses, with a slope of 0.69. *C*_max_ increased more proportionally at the lower doses (0.3 to 6.0 mg/kg) but did not increase between the highest two doses (20 and 40 mg/kg). The median terminal elimination half-lives (*t*_1/2_) were 28 to 49 h in the phase 1a SAD study and 49 to 58 h in the phase 1b MAD study. In the phase 1b MAD study (Tables 6 and 7), the *C*_max_ accumulation ratios (day 14/day 1) were approximately 2.0, 3.2, and 2.5 for 450 mg, 900 mg, and 1,125 mg, respectively, with a similar trend for AUC_0–24_ (accumulation ratios of 3.3, 4.9, and 3.5). Steady state was achieved on days 9, 12, and 11 for 450 mg, 900 mg, and 1,125 mg, respectively, which approximates 5 half-lives (Fig. 3).

**TABLE 5:** Pharmacokinetic parameters in the phase 1a SAD study^*a*^

LHF-535 dose (mg/kg)	Statistic	*C*_max_ (ng/mL)	*T*_max_ (h)	AUC_0–last_ (h · ng/mL)	AUC_0–∞_ (h · ng/mL)	*t*_1/2_ (h)	CL/F (L/h/kg)	*V*_Z_/F (L/kg)
0.3^*b*^	Mean	140	2.0	1,233	1,429	33.7	0.230	11.3
	SD	46	0.0	529	520	15.7	0.071	5.4
	CV (%)	33	0	43	36	47	31	48
	Median	123	2.0	1,171	1,300	27.9	0.231	13.2
1.0	Mean	356	2.6	3,560	3,754	30.8	0.283	11.7
	SD	89	1.2	1,017	1,094	12.3	0.069	2.4
	CV (%)	25	47	29	29	40	24	21
	Median	360	2.2	3,280	3,439	27.9	0.295	11.5
3.0	Mean	914	4.0	16,935	17,657	46.5	0.210	12.4
	SD	382	2.2	9,367	10,087	22.4	0.095	3.7
	CV (%)	42	55	55	57	48	45	30
	Median	952	4.0	13,561	13,953	38.2	0.216	12.2
6.0	Mean	1,530	2.3	29,604	29,773	49.0	0.208	14.7
	SD	394	0.8	5,509	5,571	8.7	0.043	4.3
	CV (%)	26	35	19	19	18	21	29
	Median	1,577	2.0	31,393	31,546	48.6	0.192	14.1
20	Mean	3,432	2.9	71,163	71,942	36.8	0.296	14.1
	SD	507	1.4	20,494	21,802	21.2	0.077	4.7
	CV (%)	15	47	29	30	58	26	33
	Median	3,359	3.0	68,242	68,552	29.8	0.292	13.0
40	Mean	3,388	3.0	117,042	118,233	47.4	0.355	23.6
	SD	475	1.1	27,695	28,978	12.7	0.081	6.3
	CV (%)	14	37	24	25	27	23	27
	Median	3,333	3.0	113,507	113,840	44.6	0.351	21.9

a All treatment groups comprised 6 participants. CL/F, total body clearance; *V*_Z_/F, volume of distribution; CV, coefficient of variation.

b One subject from the first cohort (0.3 mg/kg) was excluded from the calculation of AUC_0–∞_, *t*_1/2_, CL/F, and *V*_Z_/F (*n* = 5 instead of *n* = 6). The subject was excluded due to a lack of plasma concentrations to accurately define the terminal phase.

**TABLE 6:** Day 1 pharmacokinetic parameters in the phase 1b MAD study^*a*^

LHF-535 dose (mg/kg)	Statistic	*C*_max_ (ng/mL)	*T*_max_ (h)	AUC_0–τ_ (h · ng/mL)	*C*_avg_ (ng/mL)
450 mg	Mean	1,765	3	14,165	590
	SD	339	1	2,499	104
	CV (%)	19	31	18	18
	Median	1,728	4	14,629	610
900 mg	Mean	2,206	3	20,028	835
	SD	520	1	3,176	132
	CV (%)	24	37	16	16
	Median	2,051	3	19,425	809
1,125 mg^*b*^	Mean	3,060	4	31,278	1,303
	SD	951	1	10,126	422
	CV (%)	31	22	32	32
	Median	3,220	4	28,107	1,171

a All treatment groups comprised 6 participants. *C*_avg_, average concentration of drug in serum.

b Participants in the 1,125-mg group received a loading dose of 2,250 mg once on day 1, followed by a maintenance dose of 1,125 mg/day on days 2 through 14.

**TABLE 7:** Day 14 pharmacokinetic parameters in the phase 1b MAD study^*a*^

LHF-535 dose (mg/kg)	Statistic	*C*_max_ (ng/mL)	*T*_max_ (h)	AUC_0–τ_ (h · ng/mL)	*C*_avg_ (ng/mL)	AUC_0–∞_ (h · ng/mL)	*t*_1/2_ (h)	CL/F_SS_ (L/h)	V_Z_/F (L)
450 mg^*b*^	Mean	3,310	2	45,105	1,879	167,128	51	10	777
	SD	792	1	13,396	558	42,101	7	3	368
	CV (%)	24	37	30	30	25	14	32	47
	Median	2,934	2	44,059	1,836	169,744	49	10	679
900 mg	Mean	6,908	3	97,191	4,050	335,465	46	9	613
	SD	1,802	1	12,913	538	98,892	12	1	116
	CV (%)	26	37	13	13	29	25	13	19
	Median	6,560	3	95,312	3,971	353,298	51	9	623
1,125 mg^*c*^	Mean	7,544	2	108,028	4,501	281,353	58	16	1,287
	SD	2,122	1	52,485	2,187	85,112	13	2	103
	CV (%)	28	34	49	49	30	23	15	8
	Median	8,197	2	87,082	3,628	281,353	58	16	1,287

a All treatment groups comprised 6 participants. CL/F_SS_, total body clearance at steady state as calculated by dose/AUCτ.

b One subject from the first cohort (450 mg) was excluded from all summary statistics due to not receiving all 14 doses. A second subject from this cohort was excluded from the calculation of AUC_0–∞_, *t*_1/2_, CL/F_SS_, and V_Z_/F (*n* = 4 instead of *n* = 5) due to a lack of plasma concentrations to accurately define the terminal phase.

c Participants in the 1,125-mg group received a loading dose of 2,250 mg once on day 1, followed by a maintenance dose of 1,125 mg/day on days 2 through 14. Four subjects from this cohort (1,125 mg) were excluded from the calculation of AUC_0–∞_, *t*_1/2_, CL/F_SS_, and V_Z_/F (*n* = 2 instead of *n* = 6) due to a lack of plasma concentrations to accurately define the terminal phase.

## DISCUSSION

The studies described here are the first to evaluate the antiviral drug candidate LHF-535 in a human population. The phase 1a SAD study evaluated a range of single doses. The first dose (0.3 mg/kg) was selected to provide a high safety margin. Subsequent doses escalated up to 40 mg/kg, offering a broad range of exposures through which to evaluate the pharmacokinetics and safety. The phase 1b MAD study was a repeat dose design to characterize a 14-day dosing regimen. Therapeutic dosing is not generally expected to exceed this time frame. Ribavirin treatment for Lassa fever is usually provided for about 10 days (5). The third cohort in the phase 1b MAD study incorporated a loading dose (2,250 mg) as the initial dose which was twice the dose of the subsequent daily maintenance dose (1,125 mg/day). The rationale for this approach was to increase the day 1 exposure and presumably provide a more robust antiviral effect. Importantly, this approach did not produce day 1 exposures (*C*_max_ and AUC) in excess of those observed in the previous phase 1a SAD study.

LHF-535 was well tolerated in both studies. Adverse events were generally no more frequent in LHF-535 participants than in placebo participants. While there was a high frequency of gastrointestinal disorders in one cohort of the phase 1a SAD study, this was not observed in the phase 1b MAD study. There were three related SAEs in one participant revealed through telemetry monitoring, including nonconducted atrial beats, sinus bradycardia, and a long sinus pause. The weight of evidence suggests that these SAEs are likely unrelated to LHF-535: they occurred at a low dose (3.0 mg/kg), there were no similar AEs in other participants at much higher doses, and the participant exhibited other cardiac events before dosing (sinus bradycardia, a 2.8-s sinus pause, and one nonconducted atrial contraction) and also several days after dosing (three nocturnal pauses of up to 2.1 s).

LHF-535 has demonstrated efficacy in animal models of disease, including in mice (9) and guinea pigs (K. A. Cashman, submitted for publication). In the mouse model, a daily oral dose of 10 mg/kg was sufficient to protect against lethal disease, corresponding to a human dose of 0.81 mg/kg, or 61 mg/day for a 75-kg human (10). In guinea pigs, 50 mg/kg/day was sufficient to fully protect against a lethal Lassa virus challenge when initiating intraperitoneal dosing 1 or 3 days postchallenge. Systemic LHF-535 exposure using this dosing regimen in guinea pigs generated minimum plasma concentrations of 240 to 490 ng/mL and daily AUCs of 32,000 to 51,000 h·ng/mL. Taken together, these preclinical studies support the expectation that the clinical exposures described here will have therapeutic potential in a patient population. Given the good safety profile of LHF-535 at potentially therapeutic exposures in the phase 1 clinical studies, next steps include testing LHF-535 in patients with Lassa fever. Recent initiatives such as the WHO Lassa fever research and development roadmap and the West Africa Lassa Fever Consortium (WALC) have highlighted guidelines under which meaningful assessment of candidate therapeutics can be conducted for Lassa fever.

## MATERIALS AND METHODS

### Study design.

Both clinical studies were randomized, double-blind, placebo-controlled trials conducted by Nucleus Network in Melbourne, Australia. The participants were healthy male and female volunteers at least 18 but no more than 50 years of age. The primary objective of both studies was to assess the safety and tolerability of LHF-535, and the secondary objective was to evaluate LHF-535 pharmacokinetics in humans. An independent institutional review board (IRB), the Alfred Hospital Ethics Committee, approved the study protocols, and all subjects provided informed consent. The phase 1a single ascending dose (SAD) study evaluated single oral doses beginning with a weight-based dose of 0.3 mg/kg in cohort 1, with subsequent cohorts evaluating escalating doses up to 40 mg/kg. Blinded safety data through day 4 from each cohort was reviewed by a safety monitoring committee (SMC) prior to proceeding with dose escalation for the subsequent cohort. The SMC was composed of representatives from the clinical site, the sponsor, and an independent clinical monitor. The phase 1b multiple ascending dose (MAD) study evaluated 14 daily fixed oral doses beginning with 450 mg/day in cohort 1; the second cohort dose was 900 mg/day, and the third cohort received a loading dose of 2,250 mg for the first dose, followed by a daily maintenance dose of 1,125 mg/day. After each cohort, the SMC reviewed safety data through day 17 and pharmacokinetic data through day 7 prior to recommending subsequent dose escalation. All participants in both studies were screened prior to enrollment (within 28 days) and again at the clinical site check-in the day prior to dosing to verify eligibility. Subjects were fasted at least 8 h before and 4 h after all doses. Participants were discharged no less than 24 h following their final dose and returned to the clinic for scheduled follow-up visits. Participants were followed until 28 days after the final dose. Participants in each cohort in both studies were randomly assigned LHF-535 or placebo in a 3:1 ratio. The sample size was chosen based on precedent from similar investigational studies and was not based on power calculation.

### Safety assessments.

Safety assessments included adverse event monitoring, urinalysis, 12-lead ECG, vital signs, clinical chemistry, hematology, and coagulation. The phase 1a SAD study also included continuous cardiac telemetry from check-in until discharge from the clinical unit 24 h after dosing.

### Dose selection.

The starting dose for the first-in-human phase 1a SAD study was selected based on nonclinical repeat dose toxicology studies in both rodents (Sprague-Dawley rats) and nonrodents (cynomolgus macaques). The no observable adverse effect level (NOAEL) of the most sensitive species as defined by the lowest daily dose was in female rats, with a NOAEL of 50 mg/kg/day, corresponding to a human equivalent dose (10) of 8 mg/kg. The clinical starting dose of 0.3 mg/kg thus generates a safety margin over 25-fold lower than the NOAEL. Dose escalation proceeded in approximately half-log increments, following a review of all available safety and PK data, in order to evaluate doses expected to be in the therapeutically active range without exceeding the NOAEL exposures observed in nonclinical studies. The initial daily dose for cohort 1 of the phase 1b MAD was a fixed dose of 450 mg, equivalent to 6 mg/kg for a 75-kg individual. Steady-state exposure at this dose (AUC and Cmax) was projected to fall well within the range of exposures observed in the SAD study.

### Dose formulation.

LHF-535 was formulated with an inert polymer (hydroxypropylmethylcellulose acetate succinate [HPMCAS]) as a spray-dried dispersion at an LHF-535/HPMCAS ratio of 30:70. For oral dosing, the spray-dried dispersion was suspended in a compounding syrup (Ora-Blend) at a concentration of 100 mg/mL (equivalent to 30 mg/mL LHF-535).

### Pharmacokinetics.

For the phase 1a SAD study, blood samples were taken for plasma analysis predose and 0.25, 0.5, 0.75, 1, 2, 4, 8, 12, 24, 72, and 168 h following dosing, as well as on day 15 (14 days after dosing). In the phase 1b MAD study, the same 9 time points (predose through 12 h) were used, following the first (day 1) and last (day 14) doses. Additional samples were taken 24 h following each dose, immediately prior to the next day’s subsequent dose, and on days 15, 17, and 21 (24, 72, and 168 h after the last dose). Bioanalysis was performed with a validated liquid chromatography-tandem mass spectrometry (LC-MS/MS) method using deuterated LHF-535 (d12-LHF-535) as an internal standard. Pharmacokinetic parameters were calculated using a noncompartmental approach with actual sampling time points using validated software (Phoenix WinNonlin version 8.1). Terminal elimination parameters {area under the concentration-time curve extrapolated to infinity [AUC_0–∞_], terminal elimination half-life [*t*_1/2_], total body clearance [CL/F], and volume of distribution [*V*_Z_/F]} were only reported if all four criteria were met (3 or more concentrations during the log-linear portion of the terminal elimination phase, an *r*^2^ value of >0.8 for regression of the log-concentration-time data, an extrapolated AUC_0–∞_ value of <20% of total AUC_0–∞_, and a negative slope for the log regression fit). For the data presented in Fig. 1 (phase 1a SAD mean plasma concentrations), some values were below the lower limit of quantitation (1 ng/mL); in these cases, the value of 0.5 ng/mL was used to determine the average. If all samples for a given time point were below the lower limit of quantitation, the data point was not plotted.
